# Wnt signal transduction pathway and apoptosis: a review

**DOI:** 10.1186/1475-2867-10-22

**Published:** 2010-06-30

**Authors:** Nives Pećina-Šlaus

**Affiliations:** 1 Department of Biology, School of Medicine, University of Zagreb, Šalata 3, HR-10000 Zagreb, Croatia; 2 Laboratory of Neuro-oncology, Croatian Institute for Brain Research, School of Medicine University of Zagreb, Šalata 12, HR-10000 Zagreb, Croatia

## Abstract

The association between the wnt signaling pathway and apoptosis has become more firmly established in the recent scientific literature. Many reports indicate that the wnt signaling pathway regulates apoptosis through a variety of mechanisms.

The activity of wnt signaling according to specific cellular environment stimuli can either foster or restrain the processes of apoptosis. Wnt signaling regulates the early and late stages of apoptosis in both development and cellular injury in populations of neurons, endothelial cells, vascular smooth muscle cells and cardiomyocytes.

In this review I draw attention to genes and proteins of the wnt signaling pathway involved in apoptosis and describe some of their functional effects.

## Introduction

The process of apoptosis has a critical role for the survival of an organism and is functionally conserved in all higher eukaryotes, while the disregulation of apoptosis has been implicated in many different diseases. Impairment of normal apoptosis can lead to cancer or autoimmune disease [1], while too much apoptosis causes neurodegenerative and neuromuscular disease where progressive loss of neurons occurs due to apoptosis [1-3].

Association between the wnt signaling pathway and apoptosis has become increasingly established through recent reports in the literature. Genes in both the apoptotic and wnt pathways are activated in a successive coordinated fashion throughout embryonic development. Wnt signaling is essential in development because it acts as a regulator of the embryonic cell patterning, proliferation, differentiation, cell adhesion, cell survival and apoptosis. It is especially important in the development of the central nervous system because processes that include synaptic rearrangements require the expression of molecular components of the wnt pathway [4]. The pathway regulates the normal development of the neural plate and neural tube, and later of the brain, spinal cord, and numerous sensory and motor neurons [5-7]. In addition to neural tissues, wnt pathway is also critical for sound vascular and cardiac systems development [8]. Furthermore, wnt signaling modulates most aspects of osteoblast physiology, including bone cell apoptosis [9].

The mechanisms of apoptosis have proven to be crucial for homeostasis maintenance of tissues and organisms. When considering the broader picture of apoptosis, this biological phenomenon can today be interpreted in the new light as a basic mechanism in the life-preserving activities of an organism.

## Molecular key players involved in apoptosis

In mammals, two distinct apoptotic pathways exist that both end in caspase activation. The first is called the "death receptor pathway", which is initiated by extracellular ligands such as TNFalpha, or FasL (Fas ligand)/CD95L, TWEAK and TRAIL; these bind to their respective receptors on the cellular surface, TNFR, Fas/CD95, DR3, DR4/DR5.

**Figure 1:**
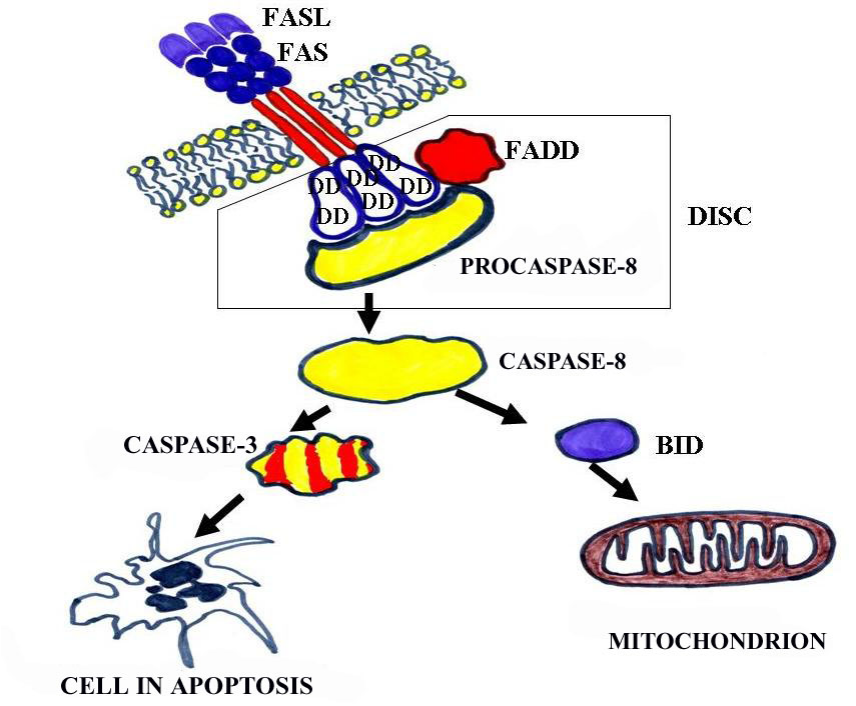
**Death receptor pathway *via *Fas ligand and Fas receptor**. DD - the specific "death domain"; DISC- death-inducing signaling complex (From: Pećina-Šlaus N. Acta Medica Croatica. Supplement., **63 **(Suppl. 2); 13-19, 2009.)

The death receptor pathway of Fas ligand and Fas receptor signaling in Figure 1 has been chosen as an example, since it represents the classical apoptotic pathway activated by extrinsic signals [10]. The proteins of the Fas ligand and Fas receptor (Fas or FasR) both act at the cytoplasmic membrane. Fas is a transmembrane protein that acts as a cell surface receptor related to the receptor, TNFR (Tumor Necrosis Factor receptor), whereas FasL is related to TNF. We have to keep in mind that the induction of apoptosis *via *death receptors is extremely fast, the ligands activating caspases within seconds of being bound to the receptor. The consequence of death ligand binding on the Fas receptor is the trimerization of receptors, which amplifies the apoptotic signal required for full triggering of the apoptotic response. Following ligand binding, a conformational change in the intracellular domain of the receptors reveals the presence of a specific domain called the "death domain" (DD). After the activation, the receptors recruit to their cytoplasmic face adaptor proteins such as FADD or TRADD. The initiator caspase-8 binds to the adaptor molecule, precisely to the "death effector domain" (DED), resulting in the formation of a casposome. The casposome is often called the "death-inducing signaling complex" (DISC) [2,3].

The second pathway of apoptotic signaling is under the control of the Bcl-2 (*B-cell lymphoma 2*) family of genes and its proteins. It is initiated by intrinsic signals transmitted via intracellular molecular components that also ultimately lead to caspase activation. Our knowledge on how this signal is transmitted is still rudimentary, but we do know that the outer membrane of mitochondria gets directly perforated as a result [1]. The complex nature of higher eukaryotes, and especially mammals, conditioned the evolution of Bcl-2 protein families with numerous members. They can be categorized as those that promote cellular survival and those that act pro-apoptotically to induce programmed cell death. More accurately, the Bcl-2 proteins should be classified into 3 categories. Proteins that promote cellular survival, *i.e*. anti-apoptotic proteins, belong to the first category. They are called Bcl-2-like survival factors and include Bcl-2, Bcl-x_L_, Bcl-w/Bcl2L2, Mcl-1, Bcl2A1/Bfl-1, NR-13, Boo/Diva/Bcl2-L-10 and Bcl-B, being similar to CD-9 in *C. elegans*. The second group is called BH3-only death factors; they act pro-apoptotically and are similar to EGL-1 in *C. elegans*. BH3-only death factors are Bik/Nbk, Blk, Hrk/DP5, BNIP3, Bcl2L11/Bim_L_/Bod, Bad, Bid, PMAIP1/Noxa, PUMA/Bbc3 and Bmf. All members of this group possess only a specific BH3 domain, a short domain consisting of 9-16 amino acids [11]. It is now believed that BH3-only proteins are the essential initiators of apoptosis.

The third category of Bcl-2 proteins is also pro-apoptotic and some scientists call them Bax-like death factors. The proteins from this group are not present in *C. elegans*. Bax, Bak, Bok/Mtd, Bcl-x_s _and Drosophila DEBCL gene belong to Bax-like death factors [2]. When apoptotic signal occurs, BH3-only proteins become active because, *inter alia*, of transcription induced by the transcription factor, p53. PUMA and Noxa are BH3-only proteins induced by p53. PUMA accounts for all the proapoptotic activity of p53 [12].

Human Bcl-2 genes are dispersed across the human genome and are not clustered at specific loci (Table 1).

**Table 1:** Chromosomal distribution of the Bcl-2 family members in the human genome.

Survival factors			Bax-like death factors			BH3 domain only death factors			
**GENE**	**LOCUS**		**GENE**	**LOCUS**		**GENE**	**LOCUS**	**GENE**	**LOCUS**

Bcl-2	18q21.33		Bak	6p21.31		Bad	11q13.1	Hrk	12q24.22

Bcl2A1 (Bfl-1)	15q25.1		Bax	19q13.33		Bcl2L11(Bim_L_/Bod)	2q13	PMAIP1 (Noxa)	18q21.32

Bcl2-L-10(Boo/DIVA)	15q21.2		Bcl-x_s_	20q11.21		Bid	22q11.21	PUMA	19q13.32

Bcl2L2 (Bcl-w)	14q11.2		Bok	2q37.3		Bik	22q13.2	BNIP3	10q26.3

Bcl-x_L_	20q11.21					Blk	8p23.1		

Mcl-1	1q21.2					Bmf	15q15.1		

**Figure 2:**
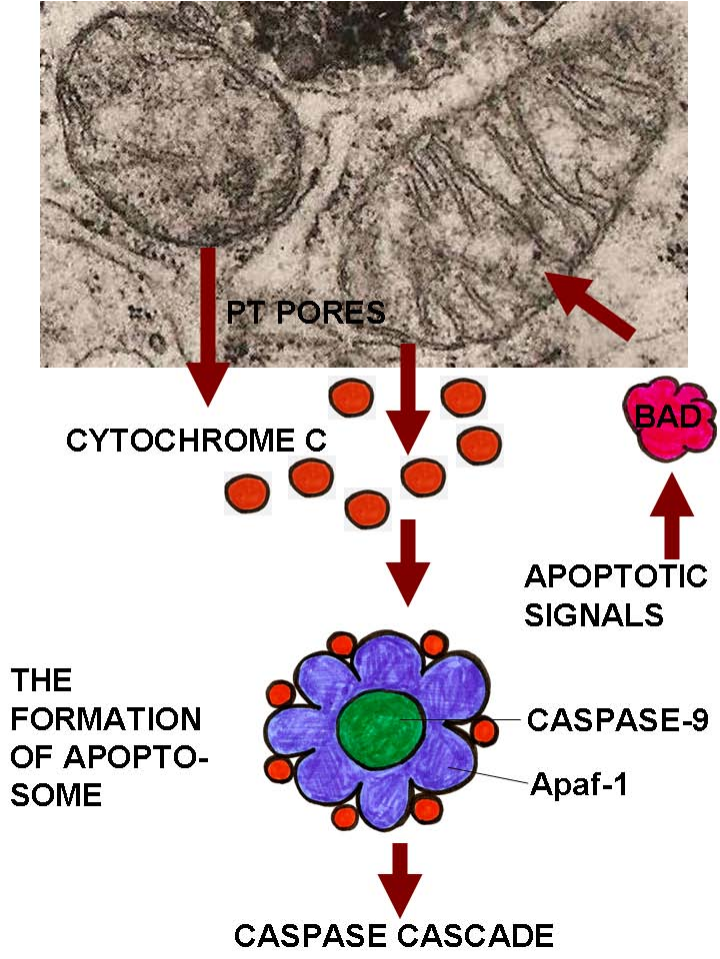
**The intrinsic pathway of apoptosis induction as implemented by mitochondria**. This figure illustrates the role of mitochondria in apoptosis.

The role of mitochondria in apoptosis is remarkable; they contain many pro-apoptotic signals of which the most important are: AIF (apoptosis inducing factor), Smac, DIABLO and definitely cytochrome C. Formation of specific mitochondrial membrane pores occurs through the action of apoptosis promoting members of the Bcl-2 protein family. These pores are called "permeability transition pores" (PT). Once the PT pores are formed, pro-apoptotic molecules - AIF, Smac, DIABLO and cytochrome C - exit the mitochondrion. The release of cytochrome C into the cytosol is especially significant event for the induction of apoptosis. Cytochrome C interacts with a protein called Apaf (the homologue of CED-4 in *C. elegans*), which in turns activates caspase-9 and later caspase-3. The multiprotein complex casposome (apoptosome) forms, conprising cytochrome C, Apaf-1, procaspase-9 and ATP [1] (Figure 2).

## Wnt signal transduction pathway and apoptosis

I will briefly describe the mechanisms of classical wnt signaling. The wnt/wingless pathway was first discovered in *Drosophila *and mouse, and is one of the most interesting signal transductions, in which key components have multiple functions. In vertebrate cells, it is named after wnt proteins, a family of highly conserved secreted signaling molecules [8]. Insights into the mechanisms of wnt action have emerged from several systems: genetics in *Drosophila *and *C. elegans*; biochemistry in cell culture; and ectopic gene expression in *Xenopus *embryos. Many wnt genes in the mouse have been mutated, leading to highly specific developmental defects. As currently understood, wnt proteins bind to receptors of the Frizzled family on the cell surface. Through several cytoplasmic relay components, the signal is transduced to β-catenin, which enters the nucleus to activate transcription of wnt target genes. Although the main signaling molecule of the pathway is β-catenin, APC is a critical component in the formation of multiprotein complex with axin/axin2, casein kinase I and glycogen synthase kinase 3-β (GSK3-β). Beta-catenin is recruited to this complex, phosphorylated, ubiquitinated and consequently heads to the proteasome. When wnt ligand is absent, β-catenin is constantly being destroyed. In response to wnt signaling, or under the circumstances of mutated APC, β-catenin is stabilized, accumulates in the cytoplasm and enters the nucleus, where it finds a partner, a member of the DNA binding protein family, the lymphoid enhancer factor-T cell factor (LEF/TCF). Together they activate the transcription programs of target genes, such as c-MYC and cyclin D1, explaining why constitutive activation of the wnt pathway can lead to cancer.

It has been well documented that wnt genes and other components of wnt signaling are critical for mammalian embryogenesis [8,9]. Furthermore, malfunctioning of this pathway in adult organism is responsible for tumorigenesis in many different tissues.

The activity of wnt signaling according to specific cellular environment stimuli can either foster or restrain the processes of apoptosis [13,14]. Wnt signaling regulates the early and late stages of apoptosis in both development and cellular injury in the cell populations of neurons, endothelial cells, vascular smooth muscle cells and cardiomyocytes [8].

Many reports indicate that the wnt signaling pathway regulates apoptosis through a variety of mechanisms. The most important mechanisms include those through wnt-BMP signaling, through SFRP2 (secreted Frizzled-related protein-2) gene expression, through β-catenin, GSK 3-β-NF-κBeta, c-Jun N-terminal kinase signaling, or gene expression of Dickkopf-1, nemo, sox 10 and tau [8,15,16].

Very interesting and novel are the data on the tumor suppressor protein, adenomatous polyposis coli (APC), and its role in apoptosis [14,17,18]. The gene encodes a large multidomain 2843 amino acid protein that is expressed in a number of fetal and adult human tissues. The wild type protein of ~310 kDa has multiple functional domains. APC is illustrative of the multiple roles that certain tumor suppressors play in a cell. The protein of this tumor suppressor gene has many cellular functions: as a component of the wnt signal transduction pathway, of adherens junctions, and the mechanism of cytoskeleton stabilization. Mutation analysis of the APC gene revealed over 400 different germline mutations responsible for familial adenomatous polyposis coli (FAP), but the overall number of detected mutations, germline and somatic [19] exceeds 700 according to the Human Gene Mutation Database [HGMD, http://www.hgmd.org]. The majority of detected mutations result in a truncated (shorter) protein product. The majority of cancer mutations occur in the mutation cluster region (MCR) located within exon 15, introducing premature stop codons. Multiple truncated proteins lacking C-terminal end are produced in such a fashion. APC's role in apoptosis regulation is dependent on whether it is comprised of full length (wild type) or truncated (mutant) proteins. Overexpression of wild type APC (310 kDa) induces apoptosis, while overexpression of mutant truncated proteins maintains an anti-apoptotic mode of action. Brocardo and Henderson [14] have recently identified APC at mitochondria and noticed that the strongest accumulation is this protein's shorter truncated polypeptide sequences. Mitochondrial localization of the endogenous APC mutants with sequence 1-1309 correlated with tumor cell survival, *i.e*. reduced apoptosis. Parallel findings indicated that overexpressed APC mutant proteins can bind to Bcl-2 proteins and increase their levels in mitochondria. It is still not clear how truncated APC proteins promote survival. APC mutants are extremely mobile and one possible explanation is that they relocate Bcl-2 from other cellular places to mitochondria. Such an increase of the amount of Bcl-2 survival factors might explain the continued survival and proliferation of cancer cells.

One of the best researched wnt proteins, protein Wnt1, has also been associated with the control of apoptotic processes [20]. Wnt-1 can inhibit apoptosis it prevents the release of cytochrome C from mitochondrion, and successively inhibits the activity of caspase-9 [8,14]. Additional research has demonstrated that wnt signaling can restrain the process of apoptosis and increase survival by activating NF-κβ or inhibiting GSK3-β. GSK3-β knockout mice die *in utero *from apoptosis of hepatocytes, while cells that lack GSK3-β genes have very low levels of NF-κβ, indicating that GSK3-β function is required for the NF-κβ-mediated survival.

## Conclusions and future perspectives

Programmed cell death plays a major physiological role in multicellular organism development and maintenance of homeostasis. Pro-and anti-apoptotic mechanisms represent exceptional potential targets for disease treatment. Therapeutics that promote cell survival by restraining apoptotic processed, as well as those that could enhance apoptosis, offer opportunities for the intelligent design of future therapeutic drugs. Restoring apoptotic activity can be a promising approach to inducing death of autoimmune and cancer cells, while therapies that arrest apoptosis in neuronal degradation would reduce neurodegenerative and neuromuscular disease.

## Competing interests

The author declares that they have no competing interests.

## Acknowledgements

This work was supported by grant 108-1081870-1905 from Ministry of Science Sports and Education, Republic of Croatia.
